# Targeted antineoplastic therapy in critically ill cancer patients: a multicenter analysis of the iCHOP registry

**DOI:** 10.1007/s00277-025-06400-3

**Published:** 2025-05-10

**Authors:** Anthea Storck, Gernot Beutel, Matthias Kochanek, Peter Schellongowski, Thomas Staudinger, Nina Buchtele, Julia Cserna, Nicole Brueder, Catherina Lueck, Tobias Liebregts, Asterios Tzalavras, Jakob Hammersen, Frank Kroschinsky, Randolf Forkert, Michael G. Kiehl, Michael von Bergwelt-Baildon, Judit Grans-Sibel, Franziska Bach, Jorge Garcia Borrega, Jan-Hendrik Naendrup, Alexander Shimabukuro-Vornhagen, Dennis A. Eichenauer, Boris Böll, for the Intensive Care in Hematologic and Oncologic Patients (iCHOP) working group

**Affiliations:** 1 Department I of Internal Medicine, Center for Integrated Oncology Aachen Bonn Cologne Düsseldorf, University Hospital Cologne, Kerpener Str. 62, 50937 Cologne, Germany; 2 Department of Hematology, Hemostasis, Oncology and Stem Cell Transplantation, Hannover Medical School, Hannover, Germany; 3 Department of Medicine I, Intensive Care Unit 13i2, Medical University of Vienna, Vienna, Austria; 4 Department of Medicine I, Hematopoietic Stem Cell Transplantation Unit, Medical University of Vienna, Vienna, Austria; 5 Hematology and Stem Cell Transplantation, University Hospital Essen, Essen, Germany; 6 Department for Hematology and Medical Oncology, Jena University Hospital, Friedrich-Schiller-University Jena, Jena, Germany; 7 Department of Internal Medicine I, University Hospital Carl Gustav Carus, Dresden, Germany; 8 Department of Internal Medicine I & II, Johanniter-Hospital Bonn, Bonn, Germany; 9 Department of Internal Medicine I, Clinic Frankfurt/Oder, Frankfurt (Oder), Germany; 10 Department of Medicine III, University Hospital, LMU Munich, Munich, Germany

**Keywords:** Cancer, Targeted therapy, Immunotherapy, Toxicity, Intensive care

## Abstract

Approximately 20% of intensive care unit (ICU) patients have cancer, and their prognosis has markedly improved in recent years. In addition to improved treatment in the ICU, this is a result of advancements in cancer therapies, including the use of targeted therapies (TTs), such as antibodies and small-molecule kinase inhibitors. Despite the increasing use of TT, there are currently no comprehensive studies examining critically ill cancer patients receiving TT in the ICU. We studied the clinical characteristics of a multicenter cohort of cancer patients who received TT in the ICU. To this end, we extracted data from the iCHOP Registry, comprising critically ill cancer patients from nine centers in Germany and Austria, and analyzed patient characteristics, cancer therapies, and survival outcomes. We then employed Cox proportional hazards regression and Kaplan‒Meier survival analyses to explore factors associated with mortality. Of the 1,762 cancer patients admitted to the ICU who were analyzed for this study, 106 patients (6%) received TT in the ICU, such as antibody-based treatments, kinase inhibitors and proteasome inhibitors. Although the TT recipients were younger, there were several pronounced high-risk features in the TT cohort, as indicated by a greater proportion of hematologic malignancies and autologous stem cell transplantation (SCT), a greater percentage of progressive disease and fewer patients in complete remission at ICU admission than in patients not receiving TT in the ICU. Despite these more pronounced risk features, TT patients had a slightly longer median OS than did the other patients according to Kaplan‒Meier analysis. The factors associated with mortality according to Cox proportional hazards regression analysis included advanced directives, disease progression, SOFA score, invasive mechanical ventilation (IMV), renal replacement therapy, and duration of ICU and hospital stay. Critically ill cancer patients receiving TT in the ICU had distinct characteristics but had comparable survival outcomes compared to patients receiving any other or no antineoplastic therapy in the ICU. While disease status at ICU admission remains crucial, the present study indicates the feasibility and potential benefits of TT in selected ICU patients.

**Supplementary Information:**

The online version contains supplementary material available at 10.1007/s00277-025-06400-3.

## Background

The admission of cancer patients to the intensive care unit (ICU) has become increasingly common in recent decades. This patient population now represents approximately 18–24% of the patients treated in medical ICUs [1, 2]. Notably, there has been a significant improvement in the prognosis of critically ill cancer patients over time [3, 4].

This improved prognosis can be attributed to different factors, including advancements in supportive care, anti-infective therapy, and ICU treatment [5]. Furthermore, the introduction of more effective cancer therapies, such as targeted therapy (TT), antibody-based therapeutics, kinase inhibitors, immune modulators, and other innovative approaches, has contributed to this improvement [6]. In hematologic malignancies, anti-CD20 antibodies (e.g. rituximab and obinutuzumab) have become standard of care in B-cell lymphomas [7, 8], the CD30-directed antibody-drug conjugate brentuximab vedotin in classic Hodgkin lymphoma [9], CD38-directed antibodies (e.g. daratumumab) and proteasome inhibitors (e.g. bortezomib) in multiple myeloma [10], and tyrosine kinase inhibitors (e.g. imatinib) in chronic myeloid leukemia and Philadelphia chromosome-positive acute lymphoblastic leukemia [11, 12]. The prognosis of some solid tumors such as lung cancer has substantially improved by incorporating checkpoint inhibitors such as pembrolizumab and atezolizumab into standard treatment [13, 14].

Within the ICU setting, chemotherapy is administered to an estimated 6–13% of cancer patients, and its use in selected cases is widely accepted [15, 16]. However, despite the growing significance of TT in cancer treatment, there is a lack of comprehensive information regarding the specific characteristics of cancer patients who undergo TT during treatment in the ICU. Notably, existing studies on TT in ICU patients have focused primarily on rare severe adverse effects [5, 17–21]. There are no comprehensive data regarding the individual characteristics, outcomes, or prognostic factors of cancer patients receiving TT in the ICU.

Therefore, the objective of this study was to evaluate the clinical characteristics of a multicenter cohort of cancer patients who received TT in the ICU. We analyzed epidemiological and clinical characteristics, details of ICU and hospital stay, specific cancer therapies and determined survival and prognostic factors.

## Methods

### Study design

The data were derived from the iCHOP Registry, an academic international prospective registry of critically ill cancer patients treated in the ICU in nine participating centers in the German language area [22].

Local investigators collected de-identified data via the OpenClinica online documentation website (OpenClinica LLC and collaborators, Waltham, MA, USA). The inclusion period was from January 1st, 2014 to December 31st, 2021. Patients were eligible if they had been diagnosed with cancer within the last five years and were admitted to either an intermediate care unit or an ICU. The more life-threatening type of cancer was reported for patients with multiple cancer types. For patients admitted to the ICU more than once, only their first ICU stay was analyzed. Patients admitted after elective surgery were excluded.

This study was performed in accordance with the principles of the Declaration of Helsinki and approved by the Institutional Review of the Medical Faculty and the University Hospital Cologne on December 18, 2013 (#13–380). All participating providers from the iCHOP registry obtained institutional review board approval from their institutions in accordance with local ethics regulations.

### Data collection

The data included age, sex, body mass index (BMI), comorbidities, reason for ICU admission, previous hematopoietic stem cell transplantation (SCT) type of cancer (hematologic or solid), remission status and information about advanced directives.

Other characteristics included reasons for ICU admission; sepsis-related organ failure assessment (SOFA) score [23] at ICU admission; respiratory support; use and dose of vasopressors; renal replacement therapy; and applied therapies comprising TT, chemotherapy, radiotherapy and combinations of these approaches. Specifically, TT and the type of action were recorded. Finally, the duration of hospital stay and duration of ICU stay were registered along with survival status.

### Statistical analysis

Patient characteristics are reported as the number (percentage) for categorical variables and as the median [interquartile range (IQR)] for continuous variables. Based on the type of cancer therapy applied in the ICU, two patient subsets were distinguished (TT: patients receiving TT; Non-TT: patients receiving either chemotherapy, radiotherapy, a combination of both or no cancer therapy at all). To compare these patient subsets, the chi-square test or, as appropriate, Fisher’s exact test was used for categorical variables, while the Kruskal‒Wallis rank sum test was used for continuous variables. Model-based multiple imputation was used to account for missing values of variables using a random forest algorithm [24].

Univariate and multivariate Cox proportional hazards regression analyses were also conducted to investigate factors associated with death before hospital discharge. Patient characteristics with a priori clinical interest were further selected using a forward and backward stepwise logistic regression [25]. The outcomes are presented as hazard ratios (HRs) and 95% confidence intervals (95% CIs).

Kaplan-Meier survival analyses were performed according to cancer therapy status in the ICU. All statistical analyses were two-tailed, and p values less than 0.05 were considered to indicate statistical significance.

All statistical analyses were performed using R (version 4.1.2, R Foundation for Statistical Computing, Vienna, Austria) and RStudio software (version 2023.03.0 + 386; https://www.R-project.org/).

## Results

### Baseline patient characteristics and cancer treatment in the ICU

A total of 1,762 cancer patients treated in the ICU were enrolled in the registry between January 1st, 2014, and December 31st, 2022 (Table 1). The median age was 62 years (interquartile range [IQR] [53;70]), and approximately two-thirds of the patients were male (63%). The vast majority had comorbidities (92%), and the median SOFA score was 7.0 (IQR 5–9). Hematologic cancer was the primary diagnosis in 57% of the patients; approximately one in four patients achieved complete remission of the underlying malignancy (24%), and 21% had not received any cancer therapy before ICU admission. Respiratory insufficiency was the most common reason for ICU admission (34%), followed by shock (16%) and infection (15%, Table 2).

**Table 1:** Baseline patient characteristics

	Targeted therapy *N* = 106^a^	No targeted therapy *N* = 1,656^a^	Overall *N* = 1,762^a^	*p* ^b^
**Baseline characteristics at hospital admission**				
Age (years)	61 (48, 68)	63 (54, 71)	62 (53, 70)	**0.028**
Male sex	68 (64%)	1,044 (63%)	1,112 (63%)	0.8
BMI	25.0 (22.5, 28.7)	24.8 (22.2, 28.5)	24.8 (22.2, 28.7)	0.7
Comorbidities (at least one)	97 (92%)	1,519 (92%)	1,616 (92%)	> 0.9
Vascular comorbidity	35 (33%)	749 (45%)	784 (44%)	**0.014**
Cardiac comorbidity	31 (29%)	537 (32%)	568 (32%)	0.5
Renal comorbidity	20 (19%)	326 (20%)	346 (20%)	0.8
Pulmonary comorbidity	23 (22%)	387 (23%)	410 (23%)	0.7
Prior hematopoietic SCT				**0.003**
Allogeneic	13 (12%)	312 (19%)	325 (18%)	
Autologous	12 (11%)	76 (5%)	88 (5%)	
None	81 (76%)	1,268 (77%)	1,349 (77%)	
Advanced directives	12 (11%)	152 (9%)	164 (9%)	0.5
**Characteristics of malignancy**				
Malignancy type				**< 0.001**
Hematologic cancer	86 (81%)	919 (55%)	1,005 (57%)	
Solid cancer	20 (19%)	737 (45%)	757 (43%)	
Malignancy specification				**< 0.001**
Acute leukemia	13 (12%)	380 (23%)	393 (22%)	
Non-Hodgkin lymphoma	44 (42%)	324 (20%)	368 (21%)	
Lung cancer	7 (7%)	150 (9%)	157 (9%)	
Genitourinary cancer	1 (1%)	118 (7%)	119 (7%)	
Myelodysplastic/myeloproliferative syndrome	17 (16%)	96 (6%)	113 (6%)	
Other solid cancer types	12 (11%)	469 (28%)	481 (27%)	
Other hematologic cancer types	12 (11%)	119 (7%)	131 (7%)	
Status at hospital admission				**< 0.001**
No prior treatment	37 (35%)	340 (21%)	377 (21%)	
Complete remission	12 (11%)	410 (25%)	422 (24%)	
Progressive disease	21 (20%)	214 (13%)	235 (13%)	
Other	36 (34%)	692 (42%)	728 (41%)	

^a^Median (IQR), n (%); ^b^Wilcoxon rank sum test; Pearson’s chi-squared test; Fisher’s exact test

BMI = body mass index, SCT = stem cell transplantation

**Fig. 1:**
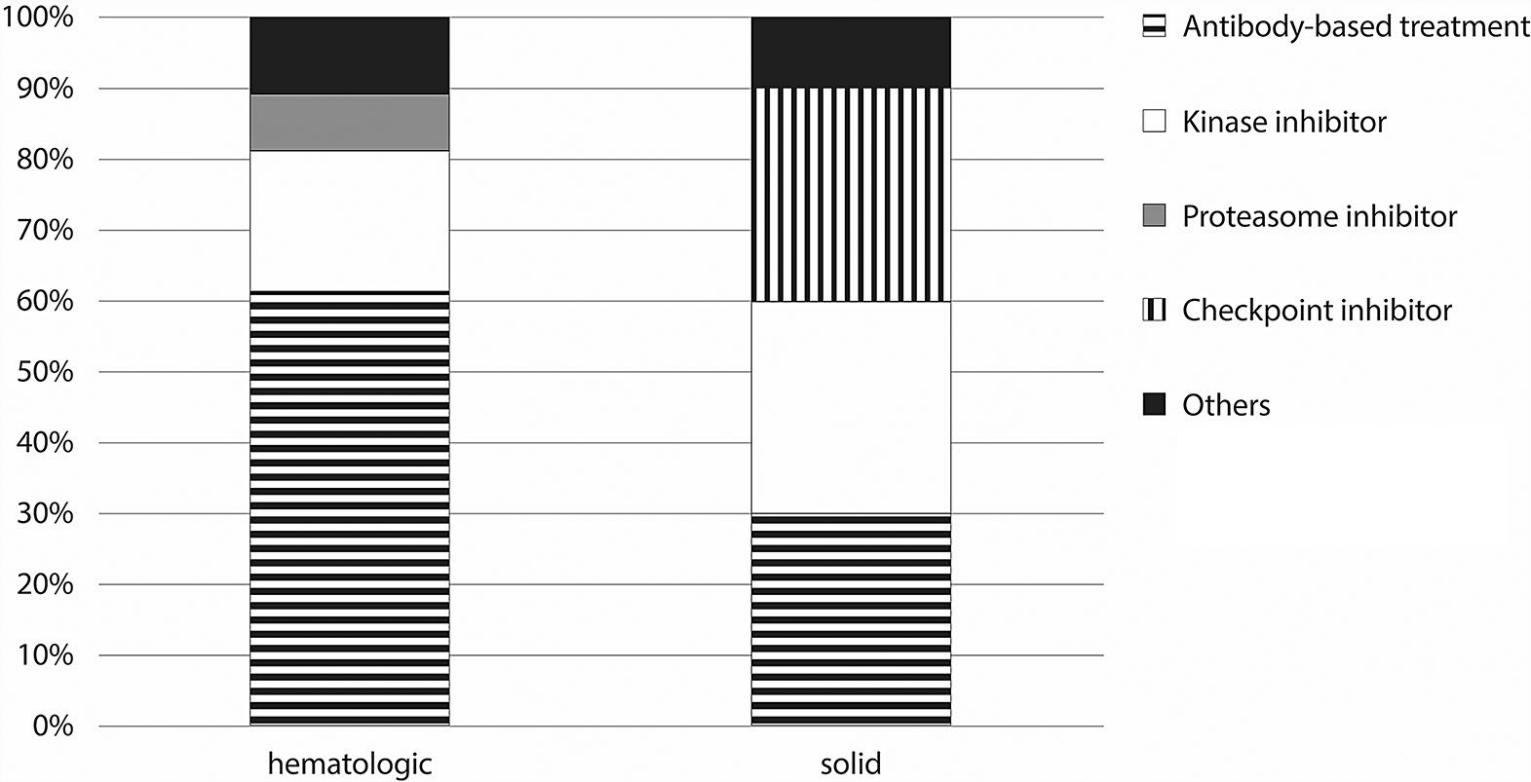
Targeted treatment agents applied in the ICU

During the ICU stay, the majority of patients (84%, *n* = 1,488) did not receive any cancer therapy. Approximately 12% of the patients (*n* = 217) were treated with chemotherapy, and radiotherapy was applied to a small subset of patients (*n* = 14; 0.8%). One hundred and six patients (6.0%) received TT in the ICU (TT cohort; Fig. 1), combined with chemotherapy in approximately half (54%, *n* = 57) and as a single treatment modality in 45% of the patients. Concerning the timepoint of inclusion during the inclusion period, there was no preference for inclusion of patients for a specific period, specifically no positively skewed distribution (data not shown).

Most TT patients (56%, *n* = 59) were treated with an antibody, including monoclonal antibodies and immunoconjugates. Kinase inhibitors were applied in 22% of the patients, and the remaining patients in the TT cohort were treated with different types of TT, including proteasome inhibitors (6.6%) and immune checkpoint inhibitors (5.7%). As expected, the type of TT varied depending on the underlying malignancy (Supplemental Fig. 1).

Compared to those in the overall cohort, the patients who received TT in the ICU were younger (median age: 60 years [IQR [48;68]) and were more frequently diagnosed with hematologic malignancies, particularly non-Hodgkin lymphoma (NHL, Table 2). Accordingly, approximately one in four patients in the TT cohort (23%, *n* = 25) had undergone prior hematopoietic stem cell transplantation (SCT), with a greater proportion of autologous SCT than in the cohort without TT (11% versus 4.6% for TT and Non-TT, respectively; *p* = 0.003). Notably, approximately 35% of the patients in the TT cohort had not received any cancer-directed treatment prior to ICU admission. Moreover, a greater percentage of patients in the TT cohort had progressive disease than patients in the cohort without TT (20% compared to 13%, respectively), and fewer patients were in complete remission at ICU admission (11% compared to 25%, respectively; Table 2).

**Table 2:** Details on admission to the ICU, treatment on the ICU and outcome

	Targeted therapy *N* = 106^a^	No targeted therapy *N* = 1,656^a^	Overall *N* = 1,762^a^	*p* ^b^
**Details of ICU admission, management and cancer therapy**				
Reason for ICU admission				**0.006**
Respiratory	34 (32%)	564 (34%)	598 (34%)	
Shock	9 (8.5%)	272 (16%)	281 (16%)	
Infection	16 (15%)	251 (15%)	267 (15%)	
Neurologic	22 (21%)	168 (10%)	190 (11%)	
Other	25 (24%)	401 (24%)	426 (24%)	
SOFA score	8.0 (5.2, 9.0)	7.0 (5.0, 9.0)	7.0 (5.0, 9.0)	0.3
Respiratory support				
Highflow oxygen	22 (21%)	379 (23%)	401 (23%)	0.6
Non invasive ventilation	33 (31%)	423 (26%)	456 (26%)	0.2
Invasive ventilation	65 (61%)	901 (54%)	966 (55%)	0.2
Vasopressors	78 (74%)	1,213 (73%)	1,291 (73%)	> 0.9
Renal replacement therapy	43 (41%)	407 (25%)	450 (26%)	**< 0.001**
Erythrocyte transfusions	71 (67%)	856 (52%)	927 (53%)	**0.002**
Platelet transfusions	52 (49%)	547 (33%)	599 (34%)	**< 0.001**
GCSF application	36 (34%)	213 (13%)	249 (14%)	**< 0.001**
Cancer therapy				**< 0.001**
TT only	48 (45%)	0 (0%)	48 (3%)	
TT and CTX	57 (54%)	0 (0%)	57 (3%)	
TT, CTX and RTX	1 (1%)	0 (0%)	1 (< 0.1%)	
CTX only	0 (0%)	155 (9%)	155 (9%)	
CTX and RTX	0 (0%)	4 (0.2%)	4 (0.2%)	
RTX only	0 (0%)	9 (0.5%)	9 (0.5%)	
None	0 (0%)	1,488 (90%)	1,488 (84%)	
**Hospital and ICU stay duration**				
Hospital stay (days)	40 (20, 64)	26 (11, 46)	27 (12, 47)	**< 0.001**
ICU stay (days)	15 (6, 29)	5 (2, 14)	5 (2, 15)	**< 0.001**
Hospital survival	55 (52%)	822 (50%)	877 (50%)	0.7
ICU survival	65 (61%)	1,013 (61%)	1,078 (61%)	> 0.9

^a^Median (IQR), n (%); ^b^Wilcoxon rank sum test; Pearson’s chi-squared test; Fisher’s exact test

BMI = body mass index, SCT = stem cell transplantation

ICU = intensive care unit, TT = targeted therapy, CTX = chemotherapy, RTX = radiotherapy, SOFA = sequential organ failure assessment, GCSF = granulocyte colony-stimulating factor

### Treatment in the ICU, length of ICU and hospital stay and survival status

Overall, invasive mechanical ventilation (IMV) and high-flow nasal cannula oxygen were the most common oxygenation strategies used (55% and 23%, respectively), and more than half of the patients required treatment with vasopressors during their ICU stay (73%, *n* = 1,009). Approximately one in four patients overall received renal replacement therapy in the ICU (26%, Table 2).

The proportion of patients requiring transfusion of blood products and renal replacement therapy was greater in the TT cohort than in the control cohort, and slightly more patients required IMV (Table 2). Accordingly, the median duration of ICU stay was longer in patients receiving TT than in patients receiving other cancer treatments in the ICU (15 days, IQR: 6–29 compared to 5 days, IQR: 2–14, respectively), also translating into a considerably longer median hospital stay (Table 2).

**Fig. 2:**
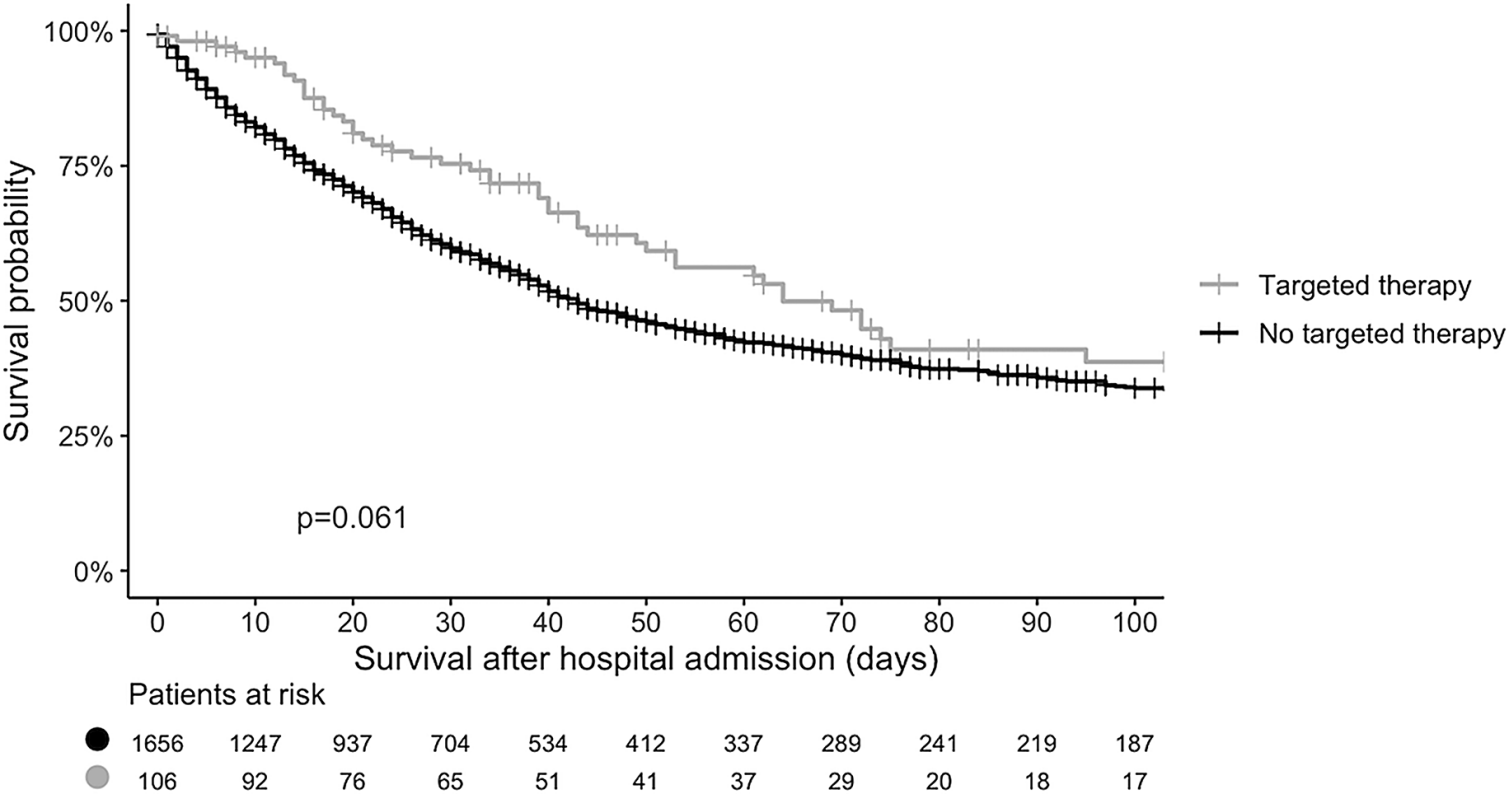
Kaplan‒Meier estimate of survival by therapy cohort

Notably, ICU survival and hospital survival were comparable in both cohorts (Table 2). According to the Kaplan–Meier analysis, the median overall survival (OS) was 44 days in the complete cohort, which was slightly longer for patients receiving TT than for patients receiving any other or no cancer treatment in the ICU (64 days compared to 43 days, respectively; *p* = 0.061; Fig. 2). Regarding subgroups, 86 and 20 patients of 106 patients who received TT had hematologic and solid cancer, respectively, and 42/86 and 13/20 of the patients with hematologic and solid cancer in the TT cohort survived, respectively.

Although not significantly different, patients who received a combination of TT and chemotherapy as cancer treatment in the ICU had longer survival than did the other patients (Supplemental Fig. 2).

### Factors associated with mortality after hospital admission

**Fig. 3:**
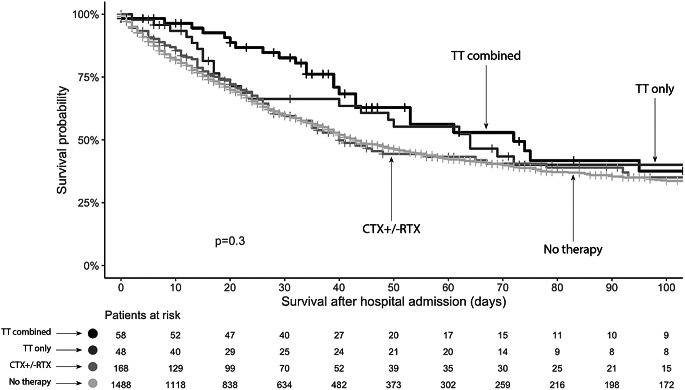
Standardized hazard ratios of factors associated with mortality after hospital admission in the overall cohort. The error bars represent the 95% confidence intervals. ICU = intensive care unit, SOFA = sequential organ failure assessment

The following factors were associated with increased mortality in patients receiving TT, as measured by the time from hospital admission to death or last follow-up in a multivariate Cox proportional hazards regression analysis: advanced directives (HR 1.85 [1.48; 2.31], *p* < 0.001), progressive disease at the time of hospital admission (HR 1.43 [1.15; 1.79], *p* = 0.002), SOFA score (HR 1.12 [1.09; 1.15], *p* < 0.001), ventilation support during the first 24 h in the ICU (significant for high-flow nasal oxygen, noninvasive ventilation, and IMV) and renal replacement therapy during the stay in the ICU (HR 1.22 [1.02; 1.47], *p* = 0.027). In addition, the duration of ICU stay (HR 1.00 [1.00; 1.01], *p* = 0.017) and length of hospital stay (HR 0.96 [0.96; 0.97], *p* < 0.001) were associated with mortality (Fig. 3). Similar results were noted in the overall cohort (supplemental figure).

Regarding the association between treatment in the ICU and mortality, there was a trend toward lower mortality in patients receiving TT in the ICU, which was most pronounced in patients receiving TT in combination with chemo- or radiotherapy (Supplemental Fig. 2).

## Discussion

To our knowledge, no comprehensive analysis of critically ill cancer patients receiving TT during their stay in the ICU has been conducted. We therefore performed a multicenter retrospective analysis addressing the characteristics and clinical course of this patient group. The major findings were as follows: (1) 6.0% of critically ill cancer patients received TT during their ICU stay; (2) patients receiving TT during their ICU stay differed from other patients with cancer treated in the ICU with regard to characteristics such as age, type of malignancy and duration of stay in the ICU and in the hospital; and (3) although not reaching statistical significance, cancer patients receiving TT during their ICU stay tended to have better overall survival than critically ill cancer patients not receiving cancer-directed treatment or non-TT during their stay in the ICU.

With a median age of 62 years and 63% male patients, the baseline characteristics in our analysis were consistent with those of a previous prevalence study from Germany investigating the frequency and characteristics of cancer patients admitted to the ICU. This earlier study comprising 1,319 patients reported a median age of 65 years and a slight male predominance (65.2%). As a previous study also included patients who were admitted for postoperative monitoring, our cohort had a greater proportion of patients with hematologic malignancies (57% in our study compared to 21.7% in the previously published prevalence study) [1].

Among the patients included in the present analysis, several characteristics differed between the general ICU cohort of patients with cancer and those who received TT. The latter were younger, more often had hematologic malignancies, were admitted to the ICU more frequently with active disease and required renal replacement therapy during their stay in the ICU in a greater proportion of patients. The 41% increase in renal replacement therapy rate in patients receiving TT may at least in part be explained by the fact that treatment of hematologic malignancies is more often associated with the necessity of renal replacement therapy due to tumor lysis syndrome. According to a French analysis including a total of 1,009 critically ill patients with hematologic malignancies, tumor lysis syndrome was identified as a major risk factor for the development of acute kidney injury, with an odds ratio of 4.66. Moreover, 26.7% of patients included in this earlier analysis required renal replacement therapy [26].

In line with the finding of more high-risk features in the TT group, such as hematologic disease and the need for renal replacement therapy, the ICU and hospital stays were longer in the TT group than in the non-TT group in the current analysis. Strikingly, the prognosis did not differ between the TT group and the non-TT group, as the ICU and hospital survival rates for the TT group were 61% and 52%, respectively, and at least equal to those of patients not receiving TT. Interestingly, a previous analysis investigating the characteristics and outcomes of a total of 110 patients with solid tumors previously treated with immune checkpoint inhibitors and admitted to the ICU thereafter indicated ICU and hospital survival rates of 79% and 73%, respectively [27]. The lower survival rate observed in the present analysis is likely due to several reasons, including a restriction to patients with solid tumors and a lower degree of illness, as indicated by the need for organ replacement therapy in the earlier analysis. For instance, vasopressors were required for 74% of the patients in the TT group in our study but only 24% of those in the previous analysis. Similarly, IMV and renal replacement therapy were more common in our cohort of patients receiving TT (61% compared to 22% and 41%, respectively, compared to 5% in patients in the TT group compared to patients in the previous analysis). A recent analysis addressed the characteristics and outcomes of patients with B-cell acute lymphoblastic leukemia (B-ALL) who developed life-threatening complications after treatment with the bispecific antibody blinatumomab. A total of 116 patients were included, 10 of whom developed life-threatening complications (cytokine release syndrome and/or immune effector cell-associated neurotoxicity syndrome or sepsis); 8 were admitted to the ICU. All but one patient admitted to the ICU survived. The total number of deaths among patients with life-threatening complications was 3 [21]. The greater ICU survival rate in comparison with that in the present analysis was likely due to different factors. These included a lower severity of illness reflected by a median SOFA score of 3.5 at ICU admission (compared to 8 in the present analysis). In addition, TT with blinatumomab was not given in the ICU for patients in the previous analysis, whereas individuals included in the present analysis had TT during their ICU stay. Finally, the low number of patients who stayed in the ICU after TT with blinatumomab, as reported in the earlier analysis, hinders a valid comparison with the data from the present study.

The present analysis demonstrated a tendency toward better survival in critically ill cancer patients receiving TT than in their counterparts not receiving TT, suggesting that TT in the ICU is feasible and beneficial for selected patients. Nonetheless, progressive disease at admission to the ICU was a negative prognostic factor for all patients with a malignancy treated in the ICU, including the subgroup of patients receiving TT. This is not unexpected, as previous analyses have indicated that progressive disease is a risk factor for increased mortality. A single-center retrospective analysis comprising 361 patients with solid tumors who had an unscheduled ICU admission between 2005 and 2013 revealed an independent association between ICU admission due to cancer progression and increased mortality after 3 and 6 months (odds ratio: 2.08 at 3 months and 2.31 at 6 months) [28]. Another analysis of 70 patients with hematologic malignancies who had undergone allogeneic SCT and were admitted to the ICU during the peri-transplant period demonstrated that progressive disease at the beginning of conditioning chemotherapy was associated with significantly worse survival outcomes than was at least stable disease at this time point [29]. Therefore, irrespective of whether patients receiving TT in the ICU have a comparably favorable outcome, disease status at admission should be taken into account when discussing therapy goals with patients and their relatives in the ICU. This is particularly important, as a response to TT typically occurs weeks after treatment; therefore, the use of TT might lead to a prolonged ICU stay and limit the possibility of time-limited ICU treatment [4, 30].

As a limitation of this analysis, some relevant information, including a detailed cancer history, previous cancer-directed therapies and causes for ICU admission, could not be obtained.

## Conclusions

This analysis provides the first comprehensive analysis of the characteristics and course of critically ill cancer patients receiving TT in the ICU. This group appears to differ from other groups of critically ill cancer patients with regard to several characteristics, whereas survival outcomes seem to be comparable.

In conclusion, the present study indicated the feasibility and potential benefits of TT in selected ICU patients.

## Electronic supplementary material

Below is the link to the electronic supplementary material.

Supplementary Material 1

## Data Availability

No datasets were generated or analysed during the current study.

## Abbreviations

BMI: Body mass index
CTX: Chemotherapy
GCSF: Granulocyte colony-stimulating factor
HR: Hazard ratio
iCHOP: International Multicenter Working Group and Registry of Intensive Care Medicine in Hematologig and Oncologic Patients
ICU: Intensive care unit
IQR: Interquartile range
RTX: Radiotherapy
SCT: Hematopoietic stem cell transplantation
SOFA-score: Sequential Organ Failure Assessment score
IMV: Invasive mechanical ventilation
