# Supplementary material for: Targeted antineoplastic therapy in critically ill cancer patients: a multicenter analysis of the iCHOP registry
Source: Ann Hematol. 2025 May 10;104(5):2937–46. doi: 10.1007/s00277-025-06400-3 (PMC12141367; doi:10.1007/s00277-025-06400-3)

**Supplemental figures**

**Supplemental Fig. 1:** Targeted treatments administered to cancer patients with hematologic or solid malignancies.


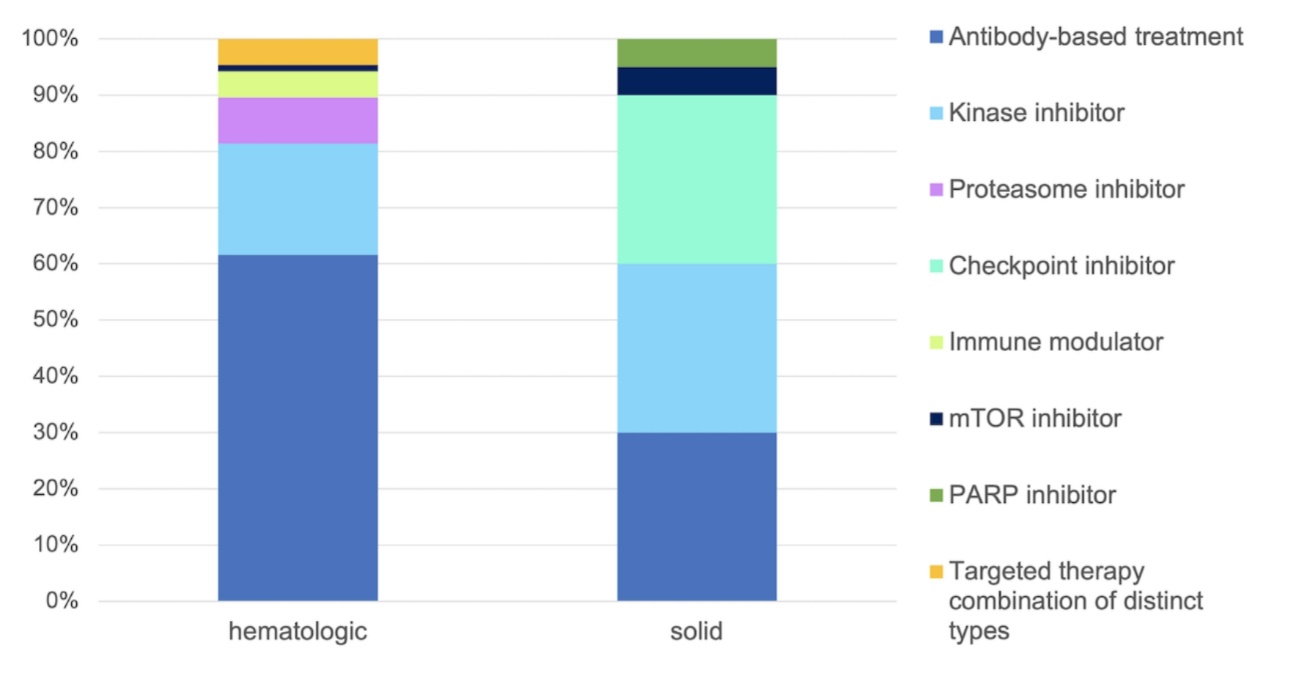


**Supplemental Fig. 2:** Kaplan‒Meier estimate of survival by therapy cohort. CTX+/-RTX = chemotherapy alone or combined with radiotherapy.


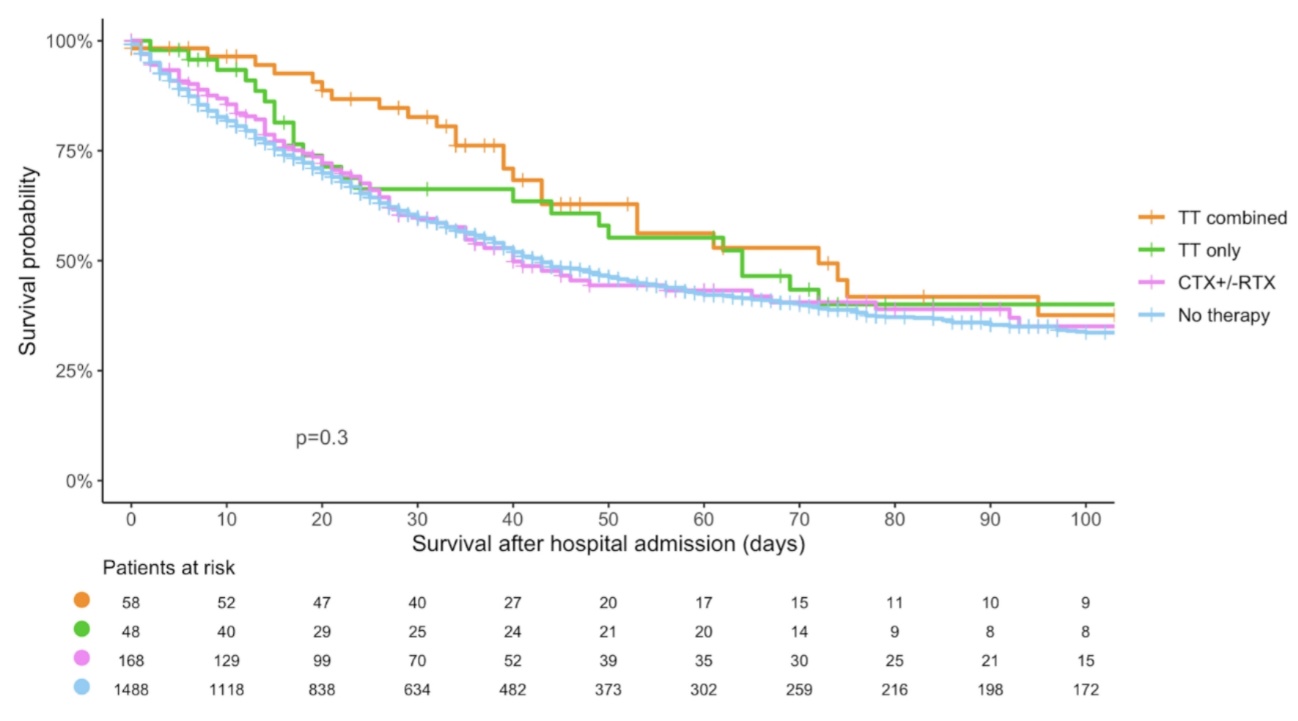


**Supplemental Fig. 3:** Standardized hazard ratios (HRs) of factors associated with mortality after hospital admission in the targeted therapy cohort. The error bars represent the 95% confidence intervals. ICU = intensive care unit, SOFA = sequential organ failure assessment.


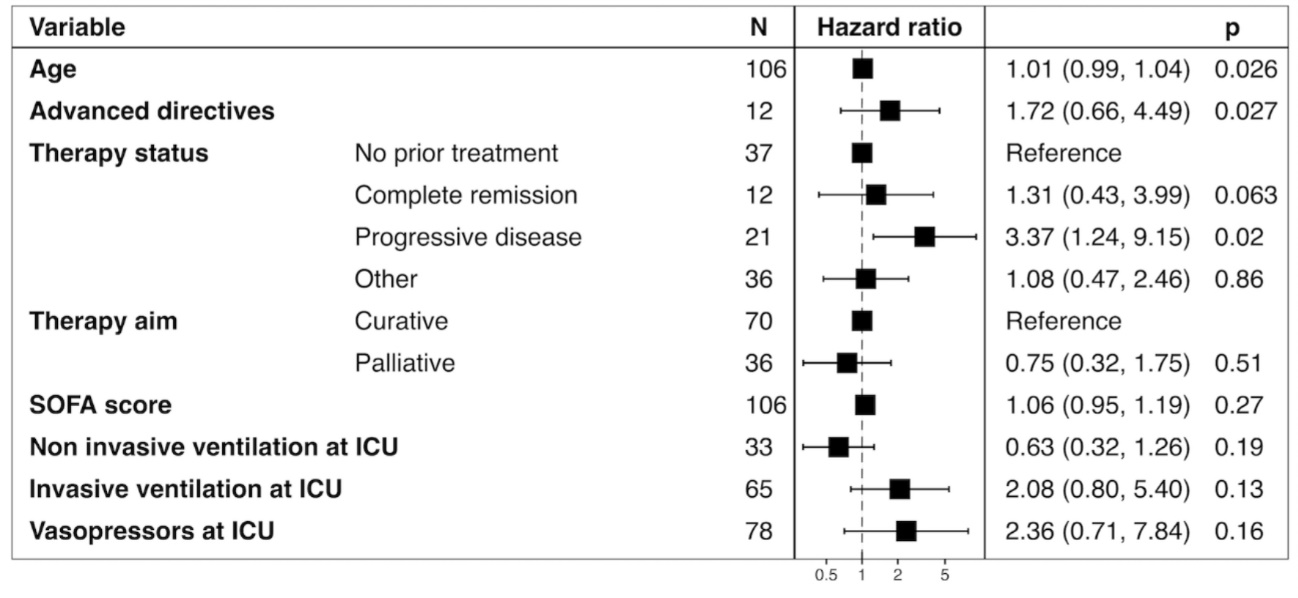

Supplement: Supplementary file 1 — Supplementary Material 1 [file 277_2025_6400_MOESM1_ESM.docx]
